# Germination Improves the Polyphenolic Profile and Functional Value of Mung Bean (*Vigna radiata* L.)

**DOI:** 10.3390/antiox9080746

**Published:** 2020-08-13

**Authors:** Garyfallia Kapravelou, Rosario Martínez, Gloria Perazzoli, Cristina Sánchez González, Juan Llopis, Samuel Cantarero, Marie Goua, Giovanna Bermano, Jose Prados, Consolación Melguizo, Pilar Aranda, María López-Jurado, Jesus M. Porres

**Affiliations:** 1 Department of Physiology, Institute of Nutrition and Food Technology (INyTA), Biomedical Research Center (CIBM), Universidad de Granada, Avda del Conocimiento s/n, 18100 Granada, Spain; kapravelou@ugr.es (G.K.); rosariomz@ugr.es (R.M.); crissg@ugr.es (C.S.G.); jllopis@ugr.es (J.L.); paranda@ugr.es (P.A.); mlopezj@ugr.es (M.L.-J.); 2 Institute of Biopathology and Regenerative Medicine (IBIMER), Biomedical Research Center (CIBM), Biosanitary Institute of Granada (IBS GRANADA), Universidad de Granada, Avda del Conocimiento s/n, 18100 Granada, Spain; gperazzoli@ugr.es (G.P.); jcprados@ugr.es (J.P.); melguizo@ugr.es (C.M.); 3 Department of Mass Spectrometry, Scientific Instrumentation Centre (CIC), Facultad de Farmacia, Universidad de Granada, Campus Universitario de Cartuja s/n, 18071 Granada, Spain; ascm@ugr.es; 4 School of Pharmacy and Life Sciences, Robert Gordon University, Aberdeen AB10 7GJ, UK; m.goua@rgu.ac.uk (M.G.); g.bermano@rgu.ac.uk (G.B.)

**Keywords:** mung bean, germination, polyphenols, antioxidant capacity, antiproliferative effect

## Abstract

The use of legumes as functional foods has gained increasing attention for the prevention and treatment of the so called non-communicable diseases that are highly prevalent worldwide. In this regard, biotechnological approaches for the enhancement of legumes’ nutritional and functional value have been extensively employed. In the present study, the process of germination increased several parameters of mung bean (*Vigna radiata* L.) functionality, including extract yield, total phenolic content and in vitro antioxidant capacity. In addition, 3-day-germinated mung bean proved to be an interesting source of dietary essential minerals and exhibited a greater variety of polyphenolic compounds compared to raw mung bean. These properties resulted in enhanced cytoprotective features of the 3-day mung bean extracts against radical oxygen species in human colorectal (HT29) and monocyte (U937) cell lines. Moreover, the antiproliferative effects were tested in different colon cancer cell lines, T84 and drug-resistant HCT-18, as well as in a non-tumor colon CCD-18 line. Altogether, our results demonstrate that the germination process improves the mung bean’s nutritional value and its potential as a functional food.

## 1. Introduction

Legumes belong to a wide group of plants (family Leguminosae) that are present as different genus and species throughout the world, and they grow under a variety of edaphic and weather conditions. Legume seeds are excellent sources of dietary essential nutrients like protein, complex carbohydrates, minerals and vitamins [1]. In addition to their nutritional value, the specific structure of their nutrients, and the presence of several bioactive components usually grouped under the generic description of non-nutritional compounds, provide legumes with beneficial properties for the management of several pathologies, and confer them great value as functional foods [2]. In particular, legumes can exert significant protection against metabolic syndrome components such as high blood pressure, hyperlipidaemia, insulin resistance and diabetes [3], as well as several types of cancer.

According to the World Health Organization (WHO), colorectal cancer is the third most frequent tumor worldwide, surpassed only by breast and lung cancer, having the second highest mortality rate [4]. This added to a survival rate of 10% after 5 years [5], and a limited treatment success with surgery or with 5-fluorouracil (5FU) combined with other agents, such as irinotecan, capecitabine or oxaliplatin, and monoclonal antibodies such as cetuximab and bevacizumab [6], emphasize the need to develop new effective strategies with therapeutic action. In recent years, a new approach for the prevention of the disease has been developed, acting on factors that increase the risk of this pathology, such as obesity and diabetes [7]. In this context, diet modulation has been suggested as a way of prevention/management against this type of tumor [8], even in combination with other cancer drugs such as 5FU [9].

Nutritional advice, mainly focusing on lifestyle changes, consists of low-fat/low-glycemic index diets and regular physical exercise [10]. This is supported by the fact that the Mediterranean diet, in which legume consumption plays a pivotal role, has been associated with reduced liver fat deposition and insulin sensitivity improvements [11]. It is well documented that legumes and legume protein hydrolysates favorably modify postprandial metabolic responses, lipid homeostasis, blood pressure dysregulations, inflammation and important glomerular function markers, as indicated from both animal and human studies [12,13,14].

Mung bean (*Vigna radiata* L.), also known as Phaseolus aureus or green gram, is a popular food legume consumed worldwide [15,16], in many different forms depending on a country’s cooking culture. It is estimated to provide substantial amounts of carbohydrates, protein, minerals, vitamins and dietary fibers, and has proved to be effective at ameliorating metabolic disorders due to the unsaturated nature of its lipid composition, its predominant insoluble fiber fraction, as well as its phenolic content strongly related to its antioxidant activity [17,18].

Germination is a biotechnological process widely used in legumes as a more economic and effective technique for enhancing the digestibility and nutritional quality of these foods in a short period of time [19,20], compared to other domestic processes such as roasting, pressure cooking and open-pan boiling [21]. Nowadays, the consumption of sprouts from legumes has increased as part of a safe and healthy diet pattern. Indeed, it has been identified that important changes in macro and micro nutrient content, as well as in bioactive components, take place during the germination process, such as increases in protein and mineral content, decreases in fat content, and the enhancement of antioxidant capacity [15,16]. The latter may be attributed to increased flavonoid, vitamin C, total and free phenolic content, which occurs during the germination process [22,23].

In view of the potential health benefits of mung bean in preventing metabolic syndrome and its related pathologies, and the established positive effects of the germination process on the nutritional and functional value of legumes, this study aimed at the following: (i) to test the influence and duration of the germination process on the antioxidant and antiproliferative capacity of mung bean extracts using different in vitro chemical assays and cell culture experiments; and (ii) to relate differences in antioxidant and antiproliferative capacity to differences in total polyphenol and mineral composition, as well as changes in polyphenolic profile induced by germination.

## 2. Materials and Methods

### 2.1. Mung Bean Seeds

Mung bean (*Vigna radiata* L., La Asturiana, León, Spain) seeds were obtained from a commercial establishment and ground to a fine powder (0.18 mm sieve) for sample analysis.

### 2.2. Germination Process

Mung bean seeds were sterilized with concentrated sodium hypochlorite for 3 min and rinsed with sterilized, type-2 water (resistivity 15 MΩ^-cm^) to remove any traces of sodium hypochlorite. They were then left to soak in water for 8 h. Afterwards, they were distributed in various trays over sheets of filter paper and left covered in darkness, at 30 °C, for 24, 48, 72 or 96 h. After this period, the sprouted seeds were lyophilized and ground to a fine powder (0.18 mm sieve) prior to analysis.

### 2.3. Acetonic Extraction and Extracts’ Preparation

The raw and germinated mung bean extracts (2-, 3- and 4-day germination) used in the initial screening stage were prepared after the extraction of mung bean flours at pH 2 for 45 min with a mixture of acetone:water:HCl (70:30:0.1) using a flour:solvent ratio of 40 mg·mL^−1^. The extraction was performed twice under the same conditions. After extraction, samples were centrifuged at 1985× *g* for 15 min and supernatant was collected. In turn, for the experiments of the analytical stage, extraction conditions were optimized to improve the extract yield and profile of polyphenolic compounds with the aim of achieving higher accuracy in the detection of antioxidant capacity and Mass Spectrometry analysis. Raw (VRR) and 3-day-germinated mung bean (VRG3D) flours were extracted twice consecutively (first with a mixture of methanol:water:HCl (70:30:0.1) and then with a mixture of acetone:water:HCl (70:30:0.1), both at pH 2, for 45 min and with a final flour:solvent ratio of 120 mg·mL^−1^). The results obtained from experiments in the screening stage provided useful information that guided the selection of the appropriate germination period for producing extracts to be used in the analytical stage. Moreover, the combined methanol and acetone extraction used in the analytical stage was selected to allow the adequate solubility of both low and high molecular weight polyphenols [24], and the detectable concentrations of polyphenols and minerals to be measured in the extracts by mass spectrometry and inductively coupled plasma mass spectrometry (ICP-MS), respectively. The supernatants from a single flour extraction process were pooled and kept at −20 °C for extraction yield, total polyphenol content and antioxidant capacity measurements. Three independent extractions were carried out per legume flour sample. Prior to each extraction and centrifugation process, N_2_ was bubbled through the samples to prevent any potential oxidation. To assess the total yield of soluble extracts, the organic solvents of samples were left to evaporate under nitrogen stream under the hood, and the evaporated samples were left subsequently to dry at 50 °C for 48 h until a constant weight was attained.

### 2.4. Determination of Total Polyphenols

Total polyphenols were determined in raw and germinated mung bean extracts using the Folin-Ciocalteu method [25]. A calibration curve based on different concentrations of gallic acid (0–600 μg·mL^−1^) was used. The results were expressed as gallic acid equivalents (mg gallic acid·mg^−1^ of extract).

### 2.5. Inhibition of Lipid Peroxidation

Thiobarbituric acid reactive substances (TBARS) were measured in rat brain homogenate as a marker of lipid peroxidation after oxidative treatment by the method of Ohkawa et al. [26] with slight modifications. Brain tissue was mixed (1:10 *w/v*) with cold 1.15% KCl/0.1% Triton X-100 with the use of a homogenizer. The mixture was centrifuged at 810× *g*, 4 °C for 25 min, and the supernatant was aliquoted and stored at −20 °C for lipid peroxidation assays. The percentage of inhibition in TBARS formation caused by the different extracts was determined as previously described by Kapravelou et al. [27].

### 2.6. Elemental Composition of Raw and 3-day Germinated Mung Bean Flours and Extracts

Determination of the magnesium (Mg), calcium (Ca), vanadium (V), manganese (Mn), iron (Fe), cobalt (Co), copper (Cu), zinc (Zn), arsenic (As) and selenium (Se) contents of raw and germinated mung bean flours and extracts was performed by ICP-MS (Agilent Technologies, Tokyo, Japan), as described by Sánchez et al. [28]. All the materials used in the analysis were previously cleaned with supra-pure nitric acid and ultrapure water (18 MΩ^-cm^, Millipore, Bedford, MA, USA). Samples were prepared by acid digestion with nitric acid and hydrogen peroxide (super-pure quality, Merck), in a microwave digester (Milestone, Sorisole, Italy). Calibration curves were prepared using stock solutions of 1000 mg·L^−1^ of each element (Merck, Darmstadt, Germany). The accuracy of the method was tested by analysis of suitable certified reference materials (BCR-191, Brown bread 0427; BCR-709, pig feed 0134; BCR-383, Haricots verts 00340). Each sample was measured three times.

### 2.7. Mass Spectrometry Analysis of Extracts’ Polyphenol Composition

High resolution mass spectrometry analysis was performed by Ultra Performance Liquid Chromatography (UPLC) coupled with a Quadrupole Time of Flight (QTOF) Mass Spectrometer (Synap G2, Waters, Milford, MA, USA) as previously reported by Martinez et al. [29]. Briefly, the analytical separation of polyphenols was performed on an Acquity HSS T33 analytical column (100 mm × 2.1 mm internal diameter, 1.8 μm; Waters, Milford, MA, USA) with a mobile phase that consisted of a gradient formed by combining deionized water with 0.5% of acetic acid as solvent A, and acetonitrile with 0.5% of acetic acid as solvent B. The flow rate of the mobile phase was 0.4 mL/min. High-resolution mass spectrometry analysis was carried out in negative electro spray ionization (ESI-ve) and spectra recorded over a 50–1200 mass/charge (*m/z*) range. All the compounds were identified based on their retention times (tR) and mass (MS) fragments using MassLynx software (Waters, Milford, MA, USA) and Chemspider database.

### 2.8. Cell Culture Experiments

HT-29 cell line, a human colorectal adenocarcinoma cell line, was obtained from the Cell Culture Resource Centre at the University of Granada, Granada, Spain. The cells were routinely cultured in 75 cm^2^ culture flasks using RPMI-1640 (Merck Life Science S.L.U, Madrid, Spain) supplemented with 10% (*v/v*) of Fetal Bovine Serum (FBS) as the culture medium. U937, a human monocyte cell line from histiocytic lymphoma, was obtained from the European Collection of Authenticated Cell Cultures (ECACC 85011440). The cells were routinely cultured in suspension in 75 cm^2^ culture flasks using RPMI-1640 supplemented with 10% (*v/v*) FBS, 100 U·mL^−1^ penicillin and 100 μg·mL^−1^ streptomycin, and 1 mM sodium pyruvate as the culture medium. T-84 (ATCC^®^ CCL-248™) and HCT-15 (ATCC^®^ CCL-225™) were obtained from the American Type Culture Collection (ATCC, Barcelona, Spain); both of these cell lines are human adenocarcinoma cell lines. CCD-18 non-tumor human colon cell line was obtained from the Scientific Instrumentation Centre (University of Granada, Granada, Spain). T-84, HCT-15 and CCD-18 cell lines were routinely cultured in 75 cm^2^ culture flasks using Dulbecco′s Modified Eagle′s Medium (DMEM-D6429, Sigma, Life Science) supplemented with 10% (*v/v*) FBS and 1% (*v/v*) Penicillin/Streptomycin as the culture medium. All cell lines were maintained at 37 °C in a humidified atmosphere of 5% CO_2_. The medium was replaced every 2–3 days.

### 2.9. Extracts’ Preparation for Cell Culture Studies

The evaporation of organic solvents from extracts (10 mL) was performed under a N_2_ flux and evaporated samples were re-suspended to original volume using 1% ethanol in Phosphate-Buffered Saline (PBS) solution. Different concentrations of extracts were studied to determine the lowest one that would not compromise cell integrity and viability (data not shown). This concentration was then combined with oxidative stressors and used for subsequent cellular assays. For cell metabolic activity and viability assays, the re-suspended extracts (10× final dilution) were mixed with RPMI-1460 medium, whereas a blank control was prepared mixing RPMI-1460 medium with ethanol (1% final concentration/well).

### 2.10. Cell Viability and Metabolic Activity Assays in HT29 Cells

Cells were seeded in two 96-well plates, at a concentration of 5 × 10^4^ cells/well, in 150 μL RPMI 10% FBS per well. Cell viability was tested using Crystal Violet assay (CV), whereas cell metabolic activity was measured using a 3-(4,5-dimethylthiazol-2yl)-2,5-diphenyl tetrazolium bromide (MTT) assay. Cells were incubated overnight, and, on the following day, the medium was changed to RPMI without FBS and incubated for 24 h, prior to performing co- or pre-incubation treatments with the different extracts and oxidative stressors. The treatment was then undertaken as follows: with a final concentration of 1 mmol·L^−1^ Paraquat (PQ)/0.7 mmol·L^−1^ S-nitroso-*N*-acetylpenicillamine (SNAP), as peroxynitrite free-radical generator [30], the cells were either pre-incubated for 16 h with 150 μL legume extracts in RPMI-1460 medium, as previously described, or with blank control, or the cells were co-incubated simultaneously with the different sample extracts or blank control. The MTT assay was performed as described by Mosmann [31] and the cell viability assay was performed as follows: after treatment, the culture medium was removed and cells were then fixed with methanol for 45 min prior to being stained with 1% (*w/v*) crystal violet solution in 50% (*v/v*) methanol for 30 min. After removing the stain, the plate was extensively rinsed with tap water. The water was fully drained and the plates were dried at 50 °C. The process was completed by adding 10 g·L^−1^ Sodium Dodecyl Sulfate (SDS) to dissolve the crystals. Absorbance was read at 595 nm using a microplate reader (Multiskan FC, Thermo Fisher Scientific, Waltham, MA, USA). Experiments were carried out 3 times with 6 replicates for each treatment in each experiment.

### 2.11. Cell Viability Assay in U937 Cells

The cell viability assay in this cell line was carried out in a similar way as described for the HT29 cell line, with slight modifications since U937 is a suspension cell line. In this case, cells were seeded in a 96-well plate, in a concentration of 2.5 × 10^4^ cells/well in 100 μL RPMI supplemented with 10% FBS, 100 U·mL^−1^ Penicillin, 100 μg·mL^−1^ Streptomycin and 1 mM Sodium Pyruvate. After leaving the plate overnight, an additional 100 µL of culture medium containing the extract and/or the corresponding treatments was added in the wells and cells were incubated for 16 h as described previously. Similarly, a 3-(4,5-dimethylthiazol-2-yl)-5-(3-carboxymethoxyphenyl)-2-(4-sulfophenyl)-2H-tetrazolium (MTS) assay was performed according to the manufacture guidelines (Sigma, Gilligham, UK). Experiments were carried out 3 times with 6 replicates for each treatment in each experiment.

### 2.12. Flow Cytometric Determination of Reactive Oxygen Species (ROS)

U937 cells were seeded in 6-well plates in a final concentration of 7 × 10^5^ cells per well in a final volume of 3 mL/well of RPMI with 10% FBS, 100 U·mL^−1^ Penicillin, 100 μg·mL^−1^ Streptomycin and 1 mM Na Pyruvate, and incubated overnight at 37 °C. The following day, cells were incubated with extracts from raw and 3-day germinated mung bean (both 10× diluted) and ethanol control (1%) for 24 h, followed by a 16 h-treatment with 1 mmol·L^−1^ PQ/0.7 mmol·L^−1^ SNAP as final concentration as previously described for the HT-29 cell line. After treatment, cells were divided into two aliquots, one for ROS determination and the other for RNA extraction and gene expression studies. The cells were washed twice with PBS by centrifugation and discarding the supernatant, prior to their incubation with 1 µM acetic 5-(chloromethyl)-2-(3,6-diacetoxy-2,7-dichloro-9H-xanthen-9-yl) benzoic anhydride (CM-H2DC-FDA) dye for 45 min at 37 °C. ROS determination was performed using a Beckman Coulter Epics XL-MCL flow cytometer (Beckman Coulter, Fullerton, CA, USA) and analysis software Expo 32 (Beckman Coulter, Fulleton, CA, USA). Experiments were carried out 3 times independently with duplicates for each treatment in each experiment.

### 2.13. Quantitative Reverse Transcription Polymerase Chain Reaction Analysis of Antioxidant Enzymes Gene Expression

U937 monocyte cells were seeded in 6-well plates at 7 × 10^5^ cells per well in a final volume of 3 mL/well of RPMI with 10% FBS, 100 U·mL^−1^ Penicillin, 100 μg·mL^−1^ Streptomycin and 1 mM Na Pyruvate for 30 h at 37 °C. The cells were then supplemented with extracts from 3-day-germinated mung bean as treatment, or ethanol as control, for 24 h. The medium including extract and ethanol was removed by collecting the cells and then centrifuging them for 5 min at 300× *g*. Subsequently, cells were resuspended in RPMI with 10% FBS, 100 U·mL^−1^ Penicillin, 100 μg·mL^−1^ and 1% Sodium Pyruvate and returned to the 6-well plates. The resuspended U937 cells were treated with 1 mM PQ and 0.7 mM SNAP, or with an equal amount of RPMI and PBS for the control and incubated for 16 h. Using TRI Reagent^®^ Solution (Invitrogen, Oxford, UK), total RNA extraction was performed following the manufacturer’s instructions. The ratio A260/A280 was undertaken to check the RNA purity, utilizing a UV/VIS spectrophotometer (ThermoSpectronic, Helios γ, Waltham, US). Reverse Transcription-PCR was performed with 100 ng RNA using Super Script III Reverse Transcriptase (Invitrogen, Oxford, UK) and random hexamers as primers (Promega, Southampton, UK) to obtain cDNA. A quantitative real time PCR (qPCR) reaction was then performed on the obtained cDNA with SYBR Green PCR Master Mix kit (Primer design, Camberley, UK), as recommended by the manufacturer. Each reaction was done in duplicate accompanied by the relevant negative control. The sequences of oligonucleotide primers for qPCR (Table 1) were designed by Doctors Bermano and Goua based on previously published data [32,33], and passed the required efficiency tests. The comparative Ct method was used to analyze the mRNA levels. The target gene expression was related to the expression of β2 microglobulin (β2M), the house keeping gene. Experiments were carried out 3 times independently with duplicates for each treatment in each experiment.

### 2.14. Cytotoxicity Screening in T84, HCT-15 and CCD-18 Cells

Cells were seeded in two 48-well plates, at a concentration of 8 × 10^3^ cells/well for T-84, 2 × 10^3^ cells/well for HCT-15 and 5 × 10^3^ cells/well for CCD-18, in 300 μL of DMEM supplemented with 10% FBS, 100 U·mL^−1^ Penicillin and 100 μg·mL^−1^ Streptomycin. Then, they were treated with raw (T-84 cell line) or 3d-germinated (T-84, HCT-15 and CCD-18 cell lines) mung bean extracts in a concentration range from 0.05 to 1 mg·mL^−1^ using dimethyl sulfoxide (DMSO) as dissolvent agent, and maintained in culture for 72 h. At the end of the incubation time, cells were fixed with 300 μL of 10% trichloroacetic acid (TCA) for 20 min at 4 °C, and stained for 20 min with 300 μL of sulforhodamine B (0.4%). After three washes with acetic acid (1%), the dye was re-suspended with 10 mM Trizmal, pH 10.5 (Merck Life Science, Madrid, Spain), and absorbance was measured at 492 nm (Titertek multiscan colorimeter, Flow Laboratories, Costa Mesa, CA, USA). Experiments were carried out 3 times with 3 replicates for each treatment in each experiment. The cytotoxicity of extracts was evaluated by comparing the relative proliferation percentage with control cells treated only with the dissolvent agent (DMSO). The percentage of cell viability was calculated using the following formula: % cell viability = [sample OD/control OD] × 100. The IC_50_ value was calculated with GraphPad Prism 6 (GraphPad Software, San Diego, CA, USA) using cell viability data.

### 2.15. Flow Cytometric Determination of Cell Cycle

T-84 cells were seeded in 6-well plates using a final concentration of 3.6 × 10^4^ cells per well in a final volume of 3 mL/well of DMEM with 10% FBS, 1% Penicillin/Streptomycin. The following day, cells were incubated with 3d-germinated mung bean extract (diluted in DMSO) with IC_50_ doses. Furthermore, the same cell cycle study was carried out with two anti-tumor drugs, such as Doxorubicin and 5-Fluorouracil, as control with IC_50_ doses (0.5 and 3.6 μM, respectively; doses were calculated by sulforhodamine B cytotoxicity assay as previously described). After 72 h, cells were collected and fixed in 70% (*v/v*) cold ethanol for 20 min. They were centrifuged (500× *g*, 5 min), rinsed once with PBS and stained with propidium iodide (PI) solution (50 μg·mL^−1^ PI, 0.5 mg·mL^−1^ RNase staining buffer) for 30 min in the dark. The samples were then analyzed using a FACScan flow cytometer combined with the Cellfit program (FACS Canto II Cytometer; BD Biosciences, San Jose, CA, USA). The PI dye binds proportionally to the amount of DNA present in the cell, which differs among G1, S and G2 phases, as does the strength of the fluorescence signal produced. Experiments were carried out 3 times with 2 replicates for each treatment in each experiment.

### 2.16. Statistical Analysis

Results are expressed as means ± standard error of the mean (SEM). Statistical significance was evaluated using Student’s *t*-test or, when appropriate, one-way analysis of variance followed by Tukey’s post-hoc test for the data of raw and 2-, 3- and 4-day-germinated mung bean extracts. Statistical analysis was performed with the Statistical Package for Social Sciences (IBM-SPSS for Windows^®^, version 22.0, Armonk, NY, USA), and differences were considered significant if *p* < 0.05.

## 3. Results

### 3.1. Screening Stage

**Figure 1:**
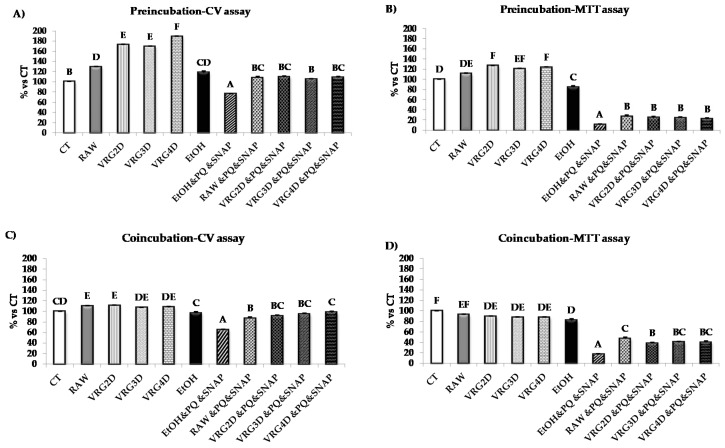
Effects of pre- or co-incubation with different mung bean extracts on viability and metabolic status of HT-29 cells treated or not with peroxynitrite. (**A**) preincubation crystal violet viability assay, (**B**) preincubation MTT metabolic status assay, (**C**) coincubation crystal violet viability assay, (**D**) coincubation MTT metabolic status assay. Results are represented as means ± SEM. Superscripts when different indicate *p* < 0.05. CT, control. RAW, raw *Vigna radiata*. VRG2D, 2d-germinated *Vigna radiata*. VRG3D, 3d-germinated *Vigna radiata*. VRG4D, 4d-germinated *Vigna radiata*. EtOH, ethanol. EtOH&PQ&SNAP, ethanol plus PQ/SNAP. RAW&PQ&SNAP, raw *Vigna radiata* plus PQ/SNAP. VRG2D&PQ&SNAP, 2d-germinated *Vigna radiata* plus PQ/SNAP. VRG3D&PQ&SNAP, 3d-germinated *Vigna radiata* plus PQ/SNAP. VRG4D&PQ&SNAP, 4d-germinated *Vigna radiata* plus PQ/SNAP. PQ, *Paraquat*. SNAP, *S-nitroso-N-acetylpenicillamine*.

In order to select the most adequate germination time for further characterization of the extracts in the analytical stage of the study, total polyphenol content and antioxidant capacity (Table 2), as well as their effects on HT29 cell viability and metabolic activity, were assessed (Figure 1A–D). Germination did not significantly affect either total polyphenol content or the antioxidant properties compared to the raw control, although the values tended to be higher in the 3-day-germinated *Vigna radiata* extract.

The effects of pre-incubating cells with extracts obtained from different germination periods prior to treatment with PQ/SNAP on cell viability and metabolic activity are shown in Figure 1A,B. As demonstrated by CV assay, cell viability was significantly enhanced (30–89%) after the addition of mung bean extracts, especially after incubation with the 4-day germination extract, which showed the highest values (89% increase) compared to CT. After the treatment with PQ and SNAP (EtOH&PQSNAP), a 34% decrease in cell viability was observed as compared to EtOH. Such decrease was prevented by pre-incubating cells with extracts of raw or germinated mung bean (Figure 1A). Pre-incubation with 1% ethanol induced a slight but significant decrease in metabolic activity compared to untreated cells (18% vs. CT). However, pre-incubation with mung bean extracts, containing the same amount of ethanol, did not induce any further decrease. On the other hand, peroxynitrite generation caused by PQ and SNAP caused a 73% decrease in the cell metabolic activity (vs. EtOH), whereas such decrease was partially prevented by pre-treatment with the different legume extracts, inducing an 11–16% improvement. No statistical differences were observed among the different extracts tested (Figure 1B).

The effects of different germination periods on cell viability and metabolic activity after co-incubation with mung bean extracts and PQ and SNAP are shown in Figure 1C,D. A significant increase (10%) in cell viability (CV assay) was observed after exposure to the different extracts compared to control cells. However, no statistically significant differences were observed between them. As expected, the addition of PQ and SNAP caused a significant reduction of 33% in cell viability, whereas co-incubation with mung bean extracts prevented such decrease and increased cell viability by 30–50%. In this assay, the 4-day germination extract demonstrated the highest values when compared to the rest of the extracts tested, however such increase was only found significant when compared with raw extract (Figure 1C). The co-incubation of the cells with the different extracts led to a 7–12% reduction in cell metabolic activity; a smaller reduction than incubation with EtOH (18%), as demonstrated by MTT assay (Figure 1D). However, the addition of PQ and SNAP significantly reduced cell metabolic activity by 65% (vs. EtOH), whereas co-incubation with mung bean extracts improved the metabolic activity of PQ- and SNAP-treated cells by 21–30% (Figure 1D).

### 3.2. Selection of Optimal Germination Conditions and Analytical Stage

Based on the anti-oxidant activity of the 3-day-germinated mung bean extract and its positive protective action from PQ- and SNAP-induced oxidative stress at the screening stage, in addition to palatability, hygiene and economic premises, this extract was selected for further testing in the analytical stage. Three days of germination is an adequate period for the large-scale preparation of sprouts, keeping good hygienic conditions and avoiding the deterioration of the organoleptic and nutritional properties of legumes that have been reported as a result of longer germination periods [19]. In addition, from an economic perspective, lengthier germination periods may not be cost-effective since the increments in bioactive potential are not proportional to the costs associated with maintaining germination periods of more than 2–3 days. Using the extracts of raw and 3-day-germinated mung bean, a series of experiments were carried out to gain more detailed knowledge on the chemical composition changes induced by germination that may affect their antioxidant capacity, and the potential molecular mechanisms involved in counteracting the detrimental effects of peroxynitrite-induced oxidative stress, as well as the antiproliferative action of the extracts tested.

#### 3.2.1. Extraction Yield, Total Polyphenol and Mineral Content of Raw and 3-day-Geminated Mung Bean Extracts

Three-day germination induced a 2.7- and 1.5-fold increase in the extraction yield and total polyphenol content of extracts, respectively, as presented in Table 3. Regarding the mineral content in flours, Mg and Ca were the minerals with higher content, followed by Fe, Zn, Mn and Cu, and by V, Co, As and Se, which were found in traces with concentrations in the range of μg·kg^−1^**.** Mineral content in flours tended to increase with germination (Table 4), and a non-statistically significant increment was especially evident for Ca, which was less pronounced in Mg, Mn, Cu, Zn, as well as in Se and Co. In contrast, there was a significant decrease in V content (*p* < 0.05). With regard to the extracts, Mg, Ca, Zn and Cu were the minerals with higher concentrations. Furthermore, the amounts of Mg and Cu, as well as those of V, Co and Se, exhibited a considerable increase in the raw *Vigna radiata* extract compared to the flour. On the other hand, germination induced a significant decrease in extract content of Mg, Cu and Zn, as well as that of Co.

The profiles of phenolic compounds obtained by mass spectrophotometry analysis found in the extracts of raw and 3-day-germinated mung bean are presented in Table 5. Six types of phenolic compounds were detected in the raw mung bean samples, whereas nine different types were detected in those that were 3d-germinated. Raw and 3-day-germinated mung bean shared several common phenolic compounds, such as astilbin, genistein, kaempferol-3-*O*-rutinase, quercetin, rutin and vitexin. However, the germination process induced the generation of new compounds, such as aspicilin, comiferylferulate, daidzein, eriodictyol, ferulic acid, glabridin, hesperetin, hesperidin, luteolin, 4-methoxycinnamic acid, naringenin, phellamurin and psoralidin.

#### 3.2.2. Bioactivity of Raw and 3-day-Geminated Mung Bean Extracts

**Figure 2:**
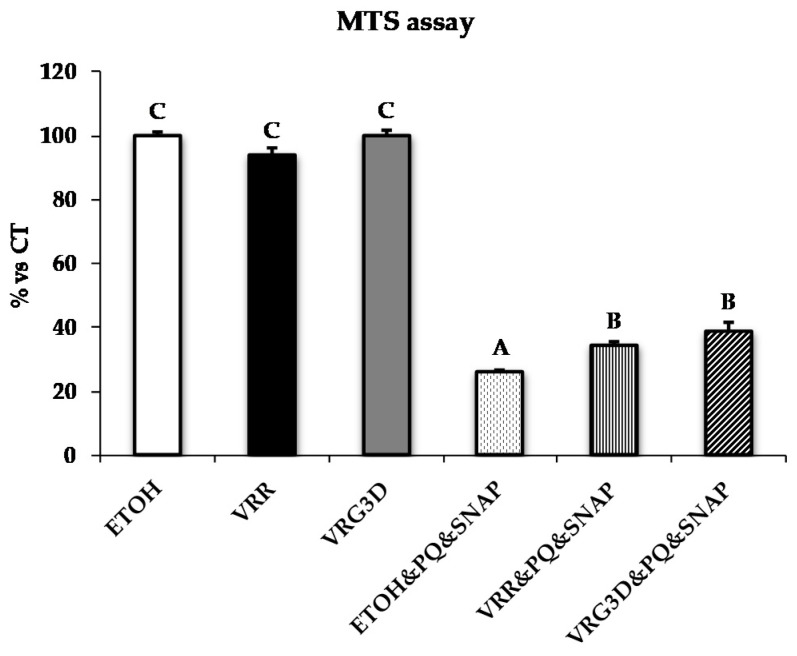
Effects of treatment with different mung bean extracts on metabolic status of U937 cells challenged with peroxynitrite. Results are represented as means ± SEM. Superscripts when different indicate *p* < 0.05. CT, control. ETOH, ethanol. VRR, raw *Vigna radiata*. VRG3D, 3d-germinated *Vigna radiata*. ETOH&PQ&SNAP, ethanol plus PQ/SNAP. VRR&PQ&SNAP, raw *Vigna radiata* plus PQ/SNAP. VRG3D&PQ&SNAP, 3d-germinated *Vigna radiata* plus PQ/SNAP. PQ, *Paraquat*. SNAP, *S-nitroso-N-acetylpenicillamine*. MTS, *3-(4,5-dimethylthiazol-2-yl)-5-(3-carboxymethoxyphenyl)-2-(4-sulfophenyl)-2H-tetrazolium*.

The effects of raw (VRR) or 3-day-germinated mung bean (VRG3D) extracts on the metabolic activity in the U937 cell line are shown in Figure 2. The addition of EtOH and/or mung bean extracts did not induce any changes in metabolic activity, whereas, as expected, the addition of peroxynitrite-generating agents caused a 72% decrease in this parameter. Treatment with extracts from VRR and VRG3D significantly improved cell metabolic activity by 8% and 13%, respectively, with a greater protective effect shown by VRG3D (vs. VRR) when compared to EtOH/PQ/SNAP treatment.

**Figure 3:**
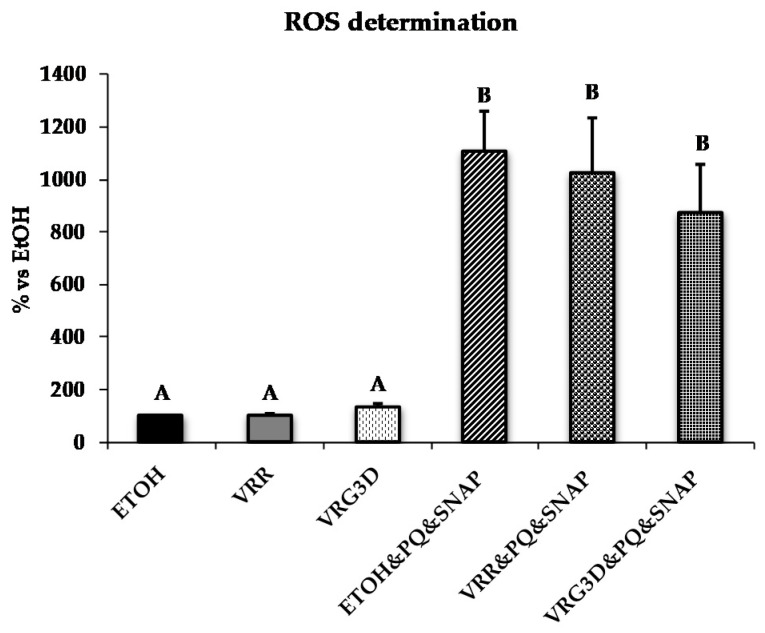
Effects of treatment with different mung bean extracts on Reactive Oxygen Species (ROS) production by U937 cells challenged with peroxynitrite. Results are represented as means ± SEM. Superscripts when different indicate *p* < 0.05. ETOH, ethanol. VRR, raw *Vigna radiata*. VRG3D, 3d-germinated *Vigna radiata*. ETOH&PQ&SNAP, ethanol plus PQ/SNAP. VRR&PQ&SNAP, raw *Vigna radiata* plus PQ/SNAP. VRG3D&PQ&SNAP, 3d-germinated *Vigna radiata* plus PQ/SNAP. PQ, *Paraquat*. SNAP, *S-nitroso-N-acetylpenicillamine*.

The effects of VRR and VRG3D extracts in counteracting ROS production induced by PQ/SNAP are presented in Figure 3. No differences were detected among treatments with EtOH and mung bean extracts, which exhibited low percentages of ROS production. In contrast, a more than 10-fold increase in ROS production was detected after the addition of PQ and SNAP. Such increase was partially reversed by raw and 3d-germinated mung bean (7% and 21%, respectively), although the differences did not achieve statistical significance.

**Figure 4:**
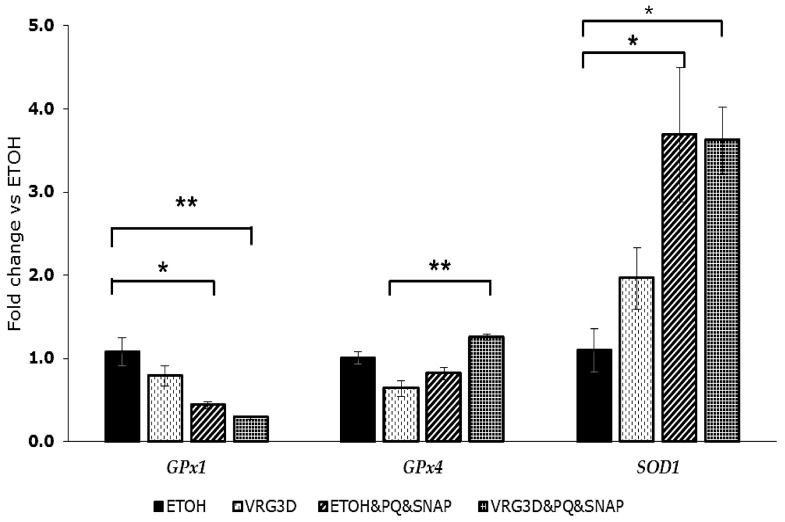
Effect of 3-day-germinated mung bean extract on antioxidant enzymes gene expression. Results are represented as means ± SEM. * indicates a significance of *p <* 0.05; ** indicates a significance of *p* < 0.01. ETOH, ethanol. VRG3D, 3d-germinated *Vigna radiata*. ETOH&PQ&SNAP, ethanol plus PQ/SNAP. VRG3D&PQ&SNAP, 3d-germinated *Vigna radiata* plus PQ/SNAP. PQ, *Paraquat*. SNAP, *S-nitroso-N-acetylpenicillamine*.

As pre-incubation with VRG3D provided slightly better protection (still not significant) against ROS production, the effect of this extract on anti-oxidant enzymes gene expression was tested and the results are presented in Figure 4.

No statistically significant differences were detected among treatments with EtOH and VRG3D extract on *GPx1, GPx4* or *SOD1* expression, even if *SOD1* gene expression doubled when cells were incubated with VRG3D. The addition of PQ and SNAP to untreated cells (EtOH vs. EtOH&PQ&SNAP) reduced *GPx1* gene expression (0.5-fold decrease *p* < 0.05), but did not affect *GPx4* gene expression, whereas it increased *SOD1* gene expression (2.5-fold, *p* < 0.05). Preincubation with VRG3D extracts did not prevent the reduction in *GPx1* gene expression (VRG3D vs. VRG3D&PQ&SNAP) induced by PQ and SNAP, whereas it induced a statistically significant increase in *GPx4* gene expression (two-fold increase, *p* < 0.01) in stressed cells. *SOD1* gene expression was not further affected by preincubation with VRG3D extract, even if a 1.5-fold increase in expression was observed in stressed cells preincubated with the extract (VRG3D&PQ&SNAP) when compared to non-stressed cells (VRG3D).

**Figure 5:**
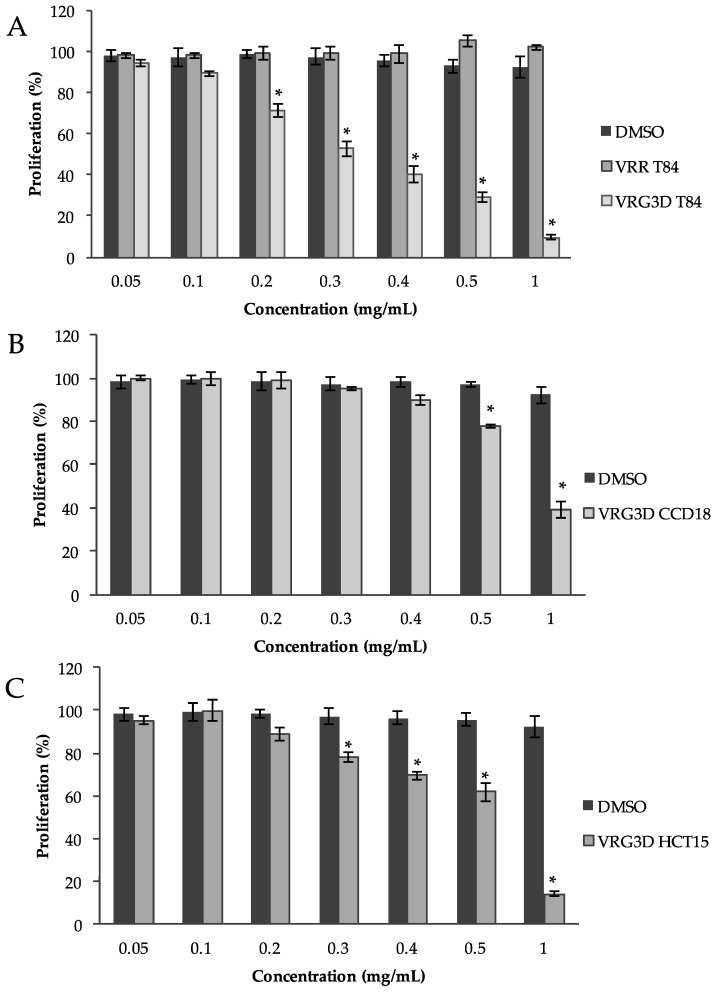
Proliferation assay in cell lines using 3d-germinated mung bean. (**A**) solvent DMSO, VRR and VRG3D mung bean extract in T-84 cell line; DMSO and VRG3D mung bean extract in CCD-18 cell line (**B**) and HCT-15 cell line (**C**). Results are expressed as % of cell survival and represented as means ± SEM. * indicates a significance of *p* < 0.05 between the treatment and control. DMSO, dimethyl sulfoxide. VRR, raw *Vigna radiata*. VRG3D, 3d-germinated *Vigna radiata*. T84, T-84 cell line. CCD18, CCD-18 cell line. HCT15, HCT-15 cell line.

Antiproliferative in vitro studies were performed using raw and 3-day-germinated mung bean extracts in a T-84 cell line (Figure 5). No cytotoxic effects were found in the T-84 cell line with VRR at the concentrations tested. However, a significant cytotoxic effect was found on this cell line after incubation with increasing concentrations of VRG3D extract (Figure 5A), obtaining an IC_50_ of 0.33 ± 0.02 mg·mL^−1^. In Figure 5B, a three-fold greater IC_50_ value of CCD-18 (0.86 ± 0.04 mg·mL^−1^) compared to the T-84 cell line indicates that that this compound can be more aggressive against tumor cells than non-tumor cells. In the case of the HCT-15 cell line, a drug-resistant cell line, the obtained IC_50_ value (0.61 ± 0.02 mg·mL^−1^) is situated in between the ones obtained for the other cell lines, due to the characteristic drug-resistance of this cell line (Figure 5C).

**Figure 6:**
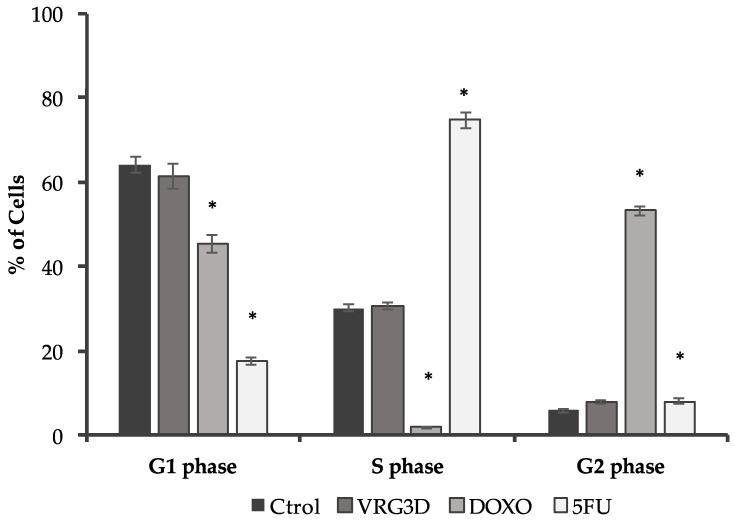
Modulation of cell cycle analysis by 3d-germinated mung bean extract in T-84 cell line. FACS scan analysis results were expressed as the percentage of labelled cells in each cell cycle phase. Results are represented as means ± SEM. CTROL, no treatment; DOXO, Doxorrubicin; 5FU, 5 Fluorouracil; VRG3D, 3d-germinated *Vigna radiata*. * indicates a significance of *p <* 0.05 between the treatment and control.

The cell cycle of the T-84 tumor cell line was studied after the addition of Doxorrubicin (DOXO), 5-Fluorouracil (5FU) or VRG3D extract using the previously determined IC_50_ values (0.27, 0.47 and 330 µg·mL^−1^, respectively). As shown in Figure 6, the cell cycle phases in the T-84 cell line without any treatment (64.1% in Growth 1 (G1), 30.1% in Synthesis (S) and 5.8% in the Growth 2/Mitosis (G2/M) phase) were modified by DOXO treatment, which induced a significant decrease in the S phase (1.8%) and a significant increase in the G2/M, in which it reached the value of 53.1%. On the other hand, 5FU treatment caused significant decrease in the G1 phase (17.4%), and an increase in the S phase (74.6%). However, no significant modification was observed in the results obtained for the treatment with VGR3D (61.4% in G1, 30.6% in S and 8% in the G2/M phase). DOXO and 5FU are known drugs for their effect in altering the cell cycle, but 3-day-germinated mung bean extract did not manage to have a similar effect.

## 4. Discussion

Functional foods are receiving a growing amount of attention due to the beneficial effects they exert on highly prevalent metabolic diseases [34] that are associated with different types of cancer [35]. Among them, legumes are of especial relevance due to their high content of bioactive phytochemicals, which have demonstrated health-promoting and/or disease-preventing potentials [36,37]. Moreover, the application of biotechnological treatments in legumes, such as germination, improves their nutritional value and enhances their health-promoting potential [23]. Even though there is an increase in antioxidant activity and phenolic content during the whole germination period, none of this follows a linear pattern [15,20], and the suggested optimal germination length varies within different species. It is noteworthy that the enhancement of total phenolic content that takes place during germination is not always correlated with higher antioxidant capacity values [15]. A possible explanation, as suggested by [38], indicates that the quality of the phenolic compounds, and not their concentration, could be responsible for the determination of the antioxidant capacity.

The extraction yield and the length of germination period are important factors affecting the antioxidant capacity. In the screening stage of the present study, no significant differences were observed during the four days of germination, whereas in the analytical stage, the different extraction process (sequential extraction with methanol and acetone) together with the higher flour:solvent ratio (40 mg·mL^−1^ in the screening vs. 120 mg·mL^−1^ in the analytical stage) resulted in a higher extraction yield of both raw and 3-day-germinated mung bean, compared to their respective yields in the screening stage. Moreover, the optimized conditions of extraction implemented for the analytical stage appeared to differentially affect the amounts of extracted compounds from the two legume flours tested, and superior yields were obtained in 3-day-germinated mung bean compared to the raw one. In other studies [39], lower values of extraction yields from raw mung bean were reported, possibly due to the different solvents and extraction ratios used. On the other hand, Guo et al. [22] reported time-dependent significant increments in vitamin C, total phenolic content and antioxidant activity even after prolonged germination periods of up to 9 days, although potential issues related to organoleptic properties, the preservation of sprouts or costs associated with such prolonged germination conditions were not discussed. In addition, the use of elicitors such as ascorbic acid during germination increased the percentage of polyphenol extraction from legume seeds [40].

The major polyphenols present in mung bean are vitexin and isovitexin [39,41]. However, under our experimental conditions, vitexin but not isovitexin was detected in raw and germinated mung bean extracts, whereas a greater number of phenolic compounds was found in our study compared to the data reported by Tang et al. [42]. The number as well as the type of phenolic compounds is highly dependent on the origin, the grain varieties and the extraction method used [43]. In this regard, vitexin, rutin and kaempferol-3-*O*-rutinose were also reported by Tang et al. [42] in germinated mung bean extracts, whereas Guo et al. [22] reported the presence of quercetin-3-*O*-glucoside in raw and germinated mung bean extracts. Although the latter bioactive compound was not found in the extracts studied, the presence of quercitrin, a glycoside formed from the flavonoid quercetin and the deoxy sugar rhamnose, was detected.

Legumes are important dietary sources of essential minerals. However, mineral availability can be compromised by the presence of non-nutritional components like phytic acid or polyphenols. Biotechnological treatments like germination or fermentation can significantly decrease or transform the above mentioned non-nutritional components, thus increasing the nutritive utilization of minerals [44,45]. The mung bean seeds used in the present experiment exhibited a high content of minerals that have been related to an adequate antioxidant status, such as Mg, Mn, Fe, Cu, Zn and minor amounts of Se. In addition, trace elements, such as V, Co or As, which are seldom reported in the literature for this legume and show some interesting biological effects in the treatment of metabolic diseases such as diabetes [46,47,48], were also found. The mineral content of mung bean used in this study differed from what has been reported in the literature, with the exception of Ca [17,49]. However, a significant variation in mineral content must be taken into consideration due to the different environmental conditions. Germination tended to increase the mineral content of mung bean in the same way as has been described for other nutrients, such as protein [50], due to the loss of dry matter, inherent to the germination process, and the subsequent concentration of certain seed components. On the other hand, the mineral contents of extracts exhibited higher amounts of the two major components in flours in a similar way to what was reported by Xu et al. [51] after the water extraction of citrus peels, with significantly higher concentrations of the main elements K, Ca and Mg followed by Cu, Fe, Mn and Zn. However, germination-induced incremental increases in the mineral contents of mung bean flour were not reflected in the higher mineral content of 3-day-germinated *Vigna radiata* extracts. This lack of correlation can be attributed to the complex changes in food matrix that take place during germination, although differences in the efficiency of mineral extraction have also been related to the plant source and solvent used [52,53], or the physicochemical conditions applied for extraction [54]. Nevertheless, the antioxidant activity in germinated extracts was significantly increased despite the lower proportion of minerals with potential antioxidant effects. This points to other factors such as total polyphenol content and profile as being responsible for their improved antioxidant capacity. Furthermore, the improvement in yield and chemical antioxidant capacity kept a close correlation with the decreased free radical formation and better metabolic status of 3-day-germinated mung bean administered to cells after intracellular peroxynitrite treatment, thus suggesting the optimized entrance of extract components into the cells, as well as enhanced antioxidant activity.

Despite the apparently weak correlation between the mineral content of extracts and their antioxidant properties, other interesting aspects related to the mineral compositions of the extracts should be highlighted. It has been widely described in the literature that the dietary pattern of the western diet, characterized by, among other things, foods with high contents of saturated fats, may not only lead to several metabolic disturbances like dyslipemia, insulin resistance or Non-alcoholic Fatty Liver Disease (NAFLD), but also to mineral imbalances and the development of osteoporosis [55]. In this regard, functional extracts like those developed in this study provide a mixture of bioactive components with potential antioxidant and anti-inflammatory properties, which may be used as mineral supplements in the treatment of bone disorders associated with the above-mentioned conditions [56]. Nevertheless, different extraction conditions may be implemented for this particular action on legume-derived functional products. In this sense, the protein hydrolyzates obtained after aqueous extraction at basic pH have been shown to exhibit an interesting polyphenolic profile and elevated mineral content [13,29]. Both of these are important features in the design of nutraceuticals that show strong bioactive potential and provide nutritionally essential elements at levels close to 10–15% of their Recommended Dietary Allowances with relatively low doses of the product.

The antioxidant effects of mung bean extracts detected using chemical methods were supported by results obtained in in vitro cell culture experiments. All the extracts tested counteracted the deleterious effects caused by peroxynitrite generation after the addition of PQ/SNAP, in both co-incubation and pre-incubation assays and in both cell lines tested; a fact that is in line with the protective action of fermented and germinated mung bean extracts against the cytotoxic effect of heat-induced oxidative stress, as shown by Yeap et al. [57]. These data reveal the protective effects against Reactive Oxygen Species that may be related to the phenolic content of mung bean extracts. In fact, the pre-treatment of lung epithelial cells with vitexin, a compound detected in the extracts used by the present study, has shown protective effects against heat-induced cytotoxicity [58]. Since ROS are highly toxic, cells may activate antioxidant enzymes in an attempt to limit this damage. In this regard, glutathione peroxidases (GPxs) and superoxide dismutases (SODs) play an important role by catalyzing the dismutation of superoxide ions (O_2_^−^) to H_2_O_2_ and O_2_, and converting H_2_O_2_ to H_2_O, respectively [59]. In our study, oxidative stress, induced by PQ and SNAP addition, decreased *GPx1* while increasing *SOD1* expression. The addition of mung bean extract to already stressed cells increased *GPx4* expression. The combination of oxidative stress and the Se-depleted conditions of the cell culture medium would explain the *GPx1* mRNA decrease, as it seems that Se status of immune cells highly affects the specific selenoprotein, which is decreased when involved in an inflammatory process, but unable to be re-synthesized due to Se deficiency [60]. The type of radical species also affects the mobilization of the different selenoproteins for their neutralization. A total of 25 selenoproteins are known [61], and *GPx1, GPx4, Sep15, Selp, Selk, Selr* and *Txnrd1* are the most abundant mRNAs detected in macrophages. GPX1 and 3 play important roles in reducing H_2_O_2_ to water, while the Se status of immune cells can directly affect the half-life of this Reactive Oxygen Species (ROS). In addition, GPX4 metabolizes phospholipid hydroperoxides, and regulates protein tyrosine phosphatase (PTP) signaling, particularly through the GPX4-mediated reduction of 12/15-lipoxygenase. On the other hand, Txnrd1 may indirectly regulate the downstream effects of H_2_O_2_ through the reduction of disulfide bonds generated by this agent in signaling molecules. Other selenoproteins such as Selp also exhibit peroxidase activity, and its antioxidant properties have been shown to affect mouse macrophage differentiation and survival during parasitic infection, although the signaling events are not clear.

Superoxides produced by PQ action can be generated through electron leakage from the electron transport chain in the mitochondria [61], and since they are electrically non-neutral, they cannot easily move out of the organelles [62]. In this regard, the incubation of cells with 3-day-germinated mung bean extract may have provided Se that was channeled to GPx4, and this could partially neutralize the superoxides produced at the mitochondrial level. Moreover, in biological systems, SOD is responsible for reducing superoxides to H_2_O_2_, which may explain the increase in this antioxidant enzyme for the protection of stressed cells [63].

There are several studies linking mung bean properties with anti-tumor activity in several tumor types, including breast [64], lung [65], liver [66] and colorectal cancer [67]. In the latter case, authors showed the antiproliferative results of raw mung bean extract (acetone/water/acetic acid, 70:29.5:0.5, v/v/v) in the SW-480 colon cancer cell line, obtaining an IC_50_ of 1.07 mg·mL^−1^, which is greater than our results in both T-84 and HCT-15 (0.33 ± 0.02 and 0.61 ± 0.02 mg·mL^−1^), respectively. However, the study of Chen et al. [68] demonstrated similar IC_50_ values of mung bean extracts in a hepatocarcinoma tumour SMMC-7721 cell line, using a protein extraction method based on isoelectric precipitation.

Regarding cell cycle results, our preliminary data for the T-84 cell line do not indicate any changes in cell cycle compared to the two exemplary anti-tumor drugs, DOXO and 5FU, which induced an arrest in phase G2/M and phase S, respectively [69,70]. However, the effect induced by the mung bean on cell cycle is controversial, as it appears to be related to the cell line used. For example, fermented mung bean caused a G0/G1 phase arrest [71] in MCF-7 breast cancer cells, as well as in a cervix cancer HeLa cell line treated with germinated mung bean extracts, but not in the liver cancer cell line HepG2 [66]. Future studies will be necessary in all cell lines to determine the involvement of our tested extracts in the cell cycle.

## 5. Conclusions

The significant improvements in extraction yield, polyphenolic content and profile, and antioxidant capacity make germinated mung bean an optimal candidate for the development of novel legume-based nutraceuticals that can be used in the combined treatment of several metabolic diseases. This higher antioxidant capacity appeared to be associated with changes in polyphenolic rather than mineral profile, and was also influenced by extraction conditions within the same germination period. The antiproliferative capacity of germinated mung bean extract in the T84 or HCT15 models of colon cancer enhances its potential as a functional ingredient, although further research is needed on the possible mechanisms responsible for this action.

## Figures and Tables

**Table 1:** Primers sequences.

Gene	Accession Number	Primer Sequence
*GPx1*	NG_012264.1	F: 5′-CACCCAGATGAACGAGCTGC-3′ R: 5′-TGTACCTGCGTAGGGGCACA-3′
*GPx4*	NG_050621.1	F: 5′-ATGCACGAGTTTTCCGCC-3′ R: 5′-CTAGAAATAGTGGGGCAGGTC-3′
*SOD1*	NG_008689.1	F: 5′-GTAAAACGACGCCAGTGAAGCCTTGTTTGAAGAGCTG-3′ R: 5′-AAACCGCGACTAACAATCAAAG-3′
*β2-microglobulin*	NG_012920.2	F: 5′-GGCTATCCAGCGTACTCCAAA-3′ R: 5′-CGGCAGGCATACTCATCTTTTT-3′

F—forward primer, R—reverse primer; GPx Glutathione Peroxidase, SOD Superoxide Dismutase.

**Table 2:** Effect of different germination periods on the total polyphenol content and antioxidant properties of mung bean extracts.

Screening Stage				
	VRR	VRG2D	VRG3D	VRG4D
Extract yield (mg·mL^−1^ extract)	7.59 ± 0.46 ^A^	8.91 ± 0.36 ^A^	8.07 ± 0.90 ^A^	9.36 ± 0.35 ^A^
Total polyphenols (μg GAE·mg^−1^ extract)	29.44 ± 2.45 ^A^	24.11 ± 1.64 ^A^	30.37 ± 0.99 ^A^	24.93 ± 1.10 ^A^
Antioxidant capacity (UAC·mg^−1^ extract)	5.41 ± 0.62 ^A^	6.12 ± 0.93 ^A^	6.41 ± 0.63 ^A^	5.65 ± 0.28 ^A^

^A^ Results are represented as means ± SEM. Means within the same row with the same superscript do not differ significantly (*p* > 0.05). VRR, raw *Vigna radiata*; VRG2D, 2-day-germinated *Vigna radiata*; VRG3D, 3-day-germinated *Vigna radiata*; VRG4D, 4-day-germinated *Vigna radiata*.

**Table 3:** Effect of 3-day-germination on extraction yield and total polyphenol content of mung bean extract.

Analytical Stage		
	VRR	VRG3D
Total yield (g extract·100 g^−1^ flour)	7.13 ± 0.23	17.53 ± 0.32 ***
Extract yield (mg extract·mL^−1^)	20.08 ± 1.08	53.35 ± 2.96 ***
Total polyphenol content (μgGAE·mg^−1^ extract)	42.13 ± 2.32	61.77 ± 3.20 ***

Results are represented as means ± SEM. *** indicates a significance of *p* < 0.001. VRR, raw *Vigna radiata*; VRG3D, 3-day-germinated *Vigna radiata*; GAE, Gallic acid equivalent.

**Table 4:** Mineral content of raw and 3d-germinated mung bean flours and extracts.

Concentration	Flours		Extracts	
	VRR	VRG3D	VRR	VRG3D
Mg (mg·kg^−1^)	1348 ± 48.9	1404 ± 94.8	4252 ± 70.6	2128 ± 37.5 **
Ca (mg·kg^−1^)	807.2 ± 15.1	938.7 ± 54.3	404.6 ± 7.8	338.7 ± 62.1
Fe (mg·kg^−1^)	48.0 ± 1.5	47.8 ± 3.9	5.36 ± 0.62	6.68 ± 0.08
Zn (mg·kg^−1^)	22.6 ± 0.8	27.5 ± 1.6	18.3 ± 0.22	9.57 ± 0.34 ***
Mn (mg·kg^−1^)	11.8 ± 0.51	13.3 ± 0.91	5.12 ± 0.08	5.05 ± 1.01
Cu (mg·kg^−1^)	7.11 ± 0.22	7.64 ± 0.50	13.3 ± 0.02	2.90 ± 0.17 ***
Se (μg·kg^−1^)	53.3 ± 8.3	96.0 ± 23.6	139.5 ± 19.3	63.0 ± 6.9
Co (μg·kg^−1^)	42.7 ± 1.7	45.3 ± 4.6	143.0 ± 1.2	73.0 ± 4.6 **
V (μg·kg^−1^)	14.7 ± 0.33	4.67 ± 1.33 *	20.0 ± 3.5	12.50 ± 1.44
As (μg·kg^−1^)	12.0 ± 0.58	10.0 ± 0.58	10.0 ± 1.15	7.50 ± 0.29

Results are represented as means ± SEM. * indicates a significance of *p <* 0.05; ** indicates a significance of *p* < 0.01; *** indicates a significance of *p* < 0.001. VRR, raw *Vigna radiata*; VRG3D, 3d-germinated *Vigna radiata*.

**Table 5:** Phenolic compounds in raw and 3-day-germinated mung bean extracts.

VRR: Phenolic Compounds in Raw Mung Bean Extracts.						
Classification	Name	Formula	Mass	Retention Time	Mass Error	Fragments
Flavanones	Astilbin	C_21_H_22_O_11_	449.108	3.01	−0.4	287.076
						235.024
						197.045
Isoflavones	Genistein	C_15_H_10_O_5_	269.045	6.2	1.1	NONE
Flavonols	Kaempferol-3-*O*-rutinose	C_27_H_30_O_15_	593.150	3.96	−0.2	431.096
						311.056
						-
	Quercitin	C_21_H_20_O_11_	447.091	3.32	−2.2	359.072
						357.060
						327.050
	Rutin	C_27_H_30_O_16_	609.145	3.5		611.161
Flavones	Vitexin	C_21_H_20_O_10_	431.097	3.67	−1.6	311.055
						283.060
						-
di-hydrochalcones	Aspalathin	C_21_H_24_O_11_	451.123	2.03	−1.3	177.018
						137.023
	Phloridzin	C_21_H_24_O_10_	435.130	4.02	1.1	315.087
						167.034
others	Taxumairol N	C_28_H_42_O_12_	569.260	4.61	0.5	527.247
						509.240
**VRG3D: Phenolic Compounds in 3-Day-germinated Mung Bean Extracts.** Common phenolic compounds between raw and germinated extracts are underlined.						
Flavanones	Astilbin	C_21_H_22_O_11_	449.107	2.92	−1.6	287.055
						269.044
	Eriodictyol	C_15_H_12_O_6_	285.055	5.18	−0.7	217.650
						161.023
						125.023
	Hesperetin	C_16_H_14_O_6_	301.070	4.76	−2.3	165.018
	Hesperidin	C_28_H_34_O_15_	609.179	4.32	−3.3	517.170
						301.070
	Naringenin	C_15_H_12_O_5_	271.060	6.12	−0.4	151.0015
						119.0505
						107.0126
Isoflavones	Genistein	C_15_H_10_O_5_	269.044	6.20	−1.1	133.029
						117.033
Flavonols	Daidzein	C_15_H_10_O_4_	253.050	5.15		-
	Kaempferol 3-*O*-β-d-rutinose	C_27_H_30_O_15_	593.150	3.96	−1.0	431.096
	Quercitin	C_21_H_20_O_11_	447.093	3.21	0.9	357.061
						285.040
						253.050
	Rutin	C_27_H_30_O_16_	609.145	3.50	−0.8	447.0921
Flavones	Vitexin	C_21_H_20_O_10_	431.097	3.65	−0.9	311.055
						283.060
	Luteolin	C_15_H_10_O_6_	285.0399	5.20	−1.1	-
Phenolic acids	Ferulic acid	C_10_H_10_O_4_	193.050	2.10	−4.7	177.055
						161.024
	4-Methoxycinnamic acid	C_10_H_10_O_3_	177.054	2.49	−1.7	147.044
						131.048
						93.034
	Aspicilin/tri-hydroxy-octadecanoic acid	C_18_H_32_O_5_	327.217	6.26	0.3	211.133
						171.102
Isoflavanes	Glabridin	C_20_H_20_O_4_	323.129	10.17	2.5	203.071
						119.049
Flavanonol	Phellamurin	C_26_H_30_O_11_	517.171	5.62	0.6	355.117
						193.086
Coumestan	Psoralidin	C_20_H_16_O_5_	335.091	9.25	−1.8	193.085
others	Comiferylferulate	C_20_H_20_O_6_	355.117	8.22	−1.1	193.086
						161.023
